# Incorporating the NICE Cambridge Prognostic Groups and Predict Prostate into a structured informed‐decision making clinic reduces over‐treatment rates of early prostate cancer

**DOI:** 10.1002/bco2.70036

**Published:** 2025-05-27

**Authors:** Vincent J. Gnanapragasam, Vineetha Thankapannair

**Affiliations:** ^1^ University of Cambridge Cambridge UK; ^2^ Cambridge University Hospitals NHS Trust Cambridge UK

**Keywords:** Cambridge Prognostic Groups, informed decision making, Predict Prostate, prostate cancer, over‐treatment

Prostate cancer poses a unique conundrum in the delivery of high‐quality care in uro‐oncology. Despite having a high prevalence, actual mortality from a diagnosis remains low.^1^ These facts mean that efforts on screening or increased PSA testing will likely result in even more cancers diagnosed for which active treatment may be unnecessary and conversely result in harm to the well‐being of an individual. Health care systems consistently neglect the fundamental fact that most clinicians are not good at estimating prognosis and treatment benefit or conveying this to patients.^2^ As a result, in the United Kingdom and elsewhere, there is considerable centre‐to‐centre and clinician‐to‐clinician variation in how prostate cancer is managed and what advice is given to patients with data from the National Prostate Cancer Audit on treatment variations clearly illustrating this fact.^3^ Informed decision‐making is critical in counselling men with newly diagnosed prostate cancer. Yet there has been (until recently) a paucity of evidence based prognostic models and individualised tools to use in informed decision making. As a result, clinicians often use their own personal judgement, prior experience and a plethora of risk models (validated and unvalidated) rather than objective data to guide their counselling.^4^ Not surprisingly, it is difficult for patients to receive consistent unbiased advice and balance benefits vs harms in decisions about how best to manage early prostate cancer. In 2021, the National Institute for Heath and Care Excellence (NICE) replaced the out‐dated 3‐tier biochemical relapse risk stratification system with the 5‐tier Cambridge Prognostic Groups (CPG).^5^ This for the first time allowed prostate cancer patients to be stratified by a model that predicted outcome based on the risk of prostate cancer mortality. The CPG prognostic model (https://cambridgeprognosticgroup.com) defines distinct subgroups of men within the old intermediate‐risk and high‐risk criteria who have very different prognostic outcomes. In head‐to‐head comparisons, the CPG model has been shown to outperform current classifiers including the EAU, AUA and NCCN models. In the same year, the Predict Prostate individualised prognostic tool (https://prostate.predict.cam) was also endorsed by NICE for informed decision making and counselling. Based on patient characteristics and clinicopathological features, Predict Prostate (video at https://www.youtube.com/watch?v=TL53pULR-94&feature=youtu.be) produces personalised prostate cancer specific and overall survival estimates displayed in a range of visual outputs to contextualise the potential benefits of radical treatment. It does not tell a patient what to do but provides an estimate of the likely gain from survival from immediate treatment versus surveillance. The use of these tools is strongly recommended by NICE, NHS England and by all major cancer charities^5^, ^6^, ^7^ and now by the EAU prostate cancer guidelines. However, to what extent the implementation of these tools can impact and reduce over‐use of radical treatment for early disease has not previously been tested.

The CPG and Predict Prostate tools were incorporated into our Multi‐Disciplinary Team (MDT) meetings in 2021 and documented in MDT outcomes and recommendations. Using these outputs, a structured counselling process was developed in a new diagnosis clinic whereby men were notified of their CPG, and for men with CPG1–3 disease, their Predict Prostate estimates. These were presented to men at the time of a new diagnosis conslutation alongside standard information on different treatment and management options. In 2022, we further added the use of the East of England Cancer Alliance ‘Know Your Options’: a website that was designed to give men direct access to NICE recommendations for specific CPG in lay language (https://www.canceralliance.co.uk/prostate). In clinic, all men were initially seen by a consultant who explained the diagnosis and prognosis, supported by a Cancer Nurse Specialist (CNS). All men also received clinic letters in which these details were included as well as QR code links to the ‘Know Your Options’ website and Predict Prostate tool (if appropriate). No decisions were requested on the same day and instead men were contacted 2–3 days later by telephone for their decision when they had time to consider the options and read the clinic letter, web‐links and information. If necessary, further CNS support was given at this time for a final decision. Once a decision was made, they were referred to the appropriate treatment specialty or enrolled into active surveillance. This structured stepwise process is shown in Figure 1, and details as well as exemplar letter is explained in the implementation toolkit in Data S1. To assess the impact, we compared decisions to choose treatment (surgery, radiotherapy, brachytherapy or any other) made by patients in 2018 versus a cohort in 2023 who were seen and counselled through this new structured clinic and process. We specifically focused on treatment selection by men with CPG1 and CPG2 disease, both of which NICE guidance recommends active surveillance as a main or equivalent option. We further retested the robustness of the observations with an additional cohort from the structured clinic from 2024 (Table 1). This study was registered as an audit with institutional approval ( ID 3579, PRN 9579).

**FIGURE 1:**
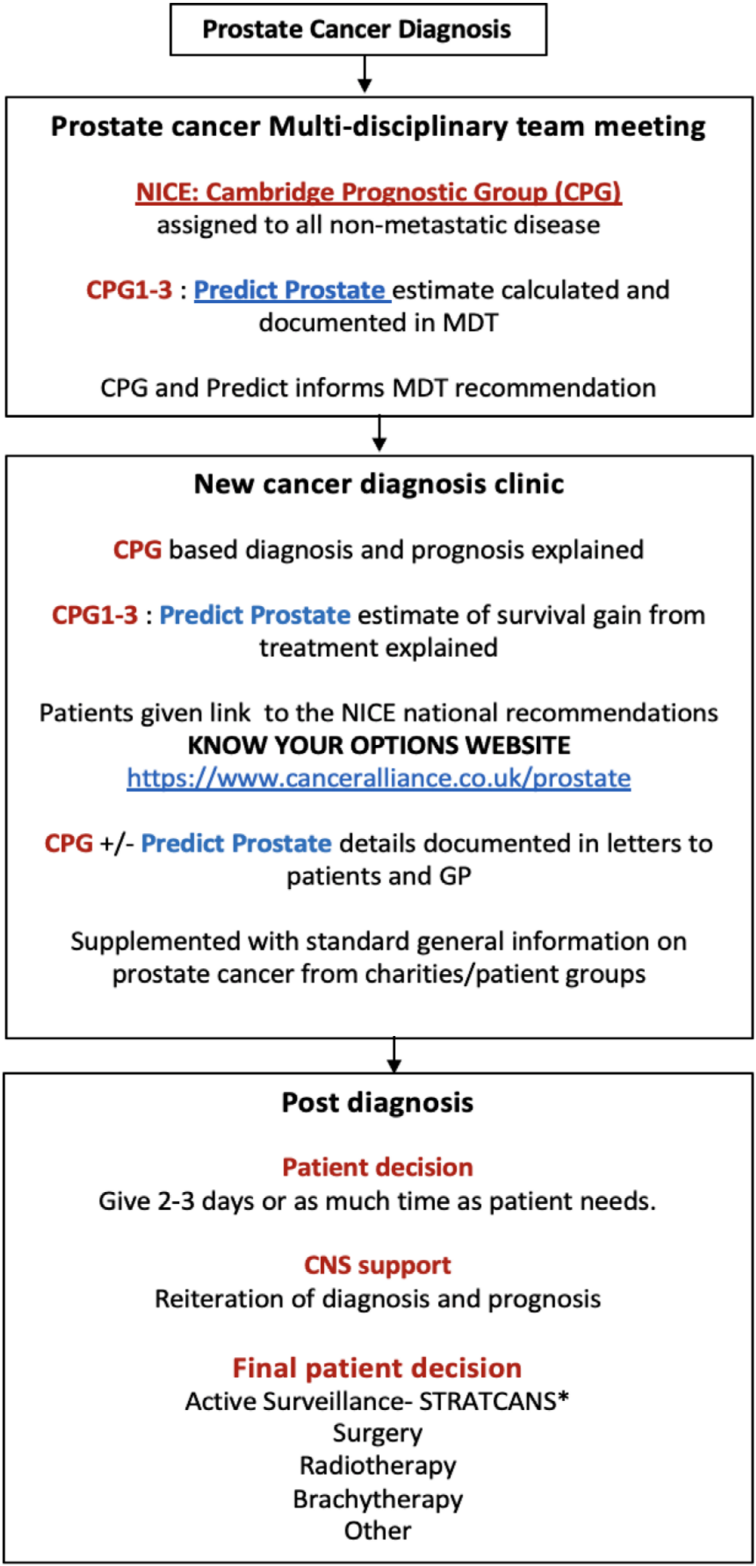
Process pathway for a structured informed decision‐making clinic for men with newly diagnosed prostate cancer. Also see implementation toolkit at https://stratcans.com/Implementation‐Toolkit‐NICE‐CPG‐Predict.pdf. *STRATCANS ‐ risk stratified active surveillance programme.

**TABLE 1:** Impact of implementing a structured counselling process in a new diagnosis clinic on rates of radical treatment selection by men diagnosed with different Cambridge prognostic group (CPG) diagnosis.

Disease category	Pre‐implementation 2018 (*n* = 177)	Post‐implementation 2023 (*n* = 138)		Post‐implementation 2024 (*n* = 111)	
	Radical treatment selected (%)	Radical treatment selected (%)	Relative change (%)	Radical treatment selected (%)	Relative change (%)
CPG1	(4/33) 12.1%	(1/33) 3.0%	−75.2%	(1/17) 5.9%	−51.2%
CPG2	(25/46) 54.3%	(12/33) 36.4%	−36.4%	(11/31) 35.5%	−34.6%
CPG3	(23/30) 76.7%	(21/24) 87.5%	+14.0%	(11/15) 73.3%	−4.4%
CPG4 and CPG5	(56/68) 82.4%	(42/48) 87.5%	+9.6%	(48/48) 100.0%	+21.3%

*Note*: Radical treatment refers to any modality of treatment with the intention of cure (surgery/radiotherapy or brachytherapy). Relative change refers to comparative change for the given year versus the rates of radical treatment in 2018.

Data from 177 men with CPG1–5 disease (non‐metastatic prostate cancer) from 2018 and 138 from 2023 were available for analysis. Another 111 men from the second validation cohort in 2024 were also included. For men diagnosed with CPG1, 12.1% selected radical treatment in 2018 versus 3.0% in 2023 representing a relative risk reduction of 75.2% (Table 1). For men with CPG2 disease, 54.3% of men in 2018 selected radical treatment versus 36.4% in 2023 (relative risk reduction of 32.9%). A similar impact was seen when the 2018 cohort was compared to men seen in 2024: CPG1 radical treatment rates were 5.9% representing a relative risk reduction of 51.2%. For men with CPG2 disease, the rate of radical treatment was 35.5% (relative risk reduction of 34.6%). In parallel, we observed an increased percentage of men with CPG4 and CPG5 disease receiving radical treatment between cohorts in 2018 versus those in 2023 and 2024 (Table 1). Men with CPG3 disease had a more mixed picture with proportionally more men receiving radical treatment in 2023 but fewer in 2024 (Table 1). These results suggest that a standardised counselling protocol incorporating unbiased, individualised prognostic and treatment benefit information can reduce the likelihood of men selecting radical treatment when diagnosed with a CPG1/CPG2 prostate cancer. These data mirror previous findings that have shown the impact of using these tools in reducing variations in treatment recommendations by clinicians and improving patient's confidence in the decision‐making process.^2^, ^8^

This work describes, to our knowledge, the first structured counselling process that incorporates modern NICE endorsed prognostic tools into real world clinical practice. We acknowledge that our data is single centre and hence limited. It is hoped however that the work published here can be used by others as a method on how to deliver a standardised counselling process and clinic. In particular we are keen that others implement, re‐test and validate our approach, potentially in a randomised trial. To this end, all the tools mentioned, CPG Predict Prostate and the Know Your Options website, are all freely available to access and use. We have further developed an implementation tool kit for any health service or clinicians who wish to replicate our process, and this is accessible at https://stratcans.com/Implementation-Toolkit-NICE-CPG-Predict-March2025.pdf (also see Data S1). We acknowledge that there may always be other confounding factors that influence patient decision making. However, one advantage of a single centre study is that we know that other mechanisms, for example, the use of clinical nurse support, what information is given, the clinic space and so on, had remained the same throughout the three periods. Hence, we feel with some confidence that it is the adoption of the structured counselling process that have been the direct and primary driver in the reduction in over‐treatment we have observed.

Standardising how patients are counselled and the information given is going to be critical to address the pandemic of over‐treatment (and under‐treatment) that has been highlighted by NPCA audits. Notwithstanding the many NHS efforts for patients to be involved in their treatment decisions, most patients still rely on their healthcare providers to guide them on management decisions, believing that their doctors/nurses must know what the real risks and benefits are and use unbiased information sources. To date, however, there has been no expert agreement or national consensus on a standard method of counselling so that men can be given the same information regardless of where they are diagnosed and seen. This is despite NICE and other national bodies placing informed and shared decision making at the heart of their guideline recommendations. Improving the current ‘wild west’ of prostate cancer counselling and information provisions is going to be crucial if efforts to improve earlier disease detection (e.g. with screening) does not simply result in a worsening of over‐treatment rates. This is also important for supporting decisions on the use of active surveillance and giving patients confidence that they fully understand their diseases prognosis and treatment decisions.

## AUTHOR CONTRIBUTIONS

**Vineetha Thankappanair**: Data collection; analysis; manuscript drafting. **Vincent J. Gnanapragasam**: Conceptualization; visualisation; methodology; project administration; supervision; manuscript drafting.

## CONFLICT OF INTEREST STATEMENT

The authors declare no conflicts of interest.

## Supporting information


**Data S1.** Implementation toolkit for the NICE Cambridge Prognostic groups and Predict Prostate
