# Supplementary material for: Incorporating the NICE Cambridge Prognostic Groups and Predict Prostate into a structured informed‐decision making clinic reduces over‐treatment rates of early prostate cancer
Source: BJUI Compass. 2025 May 27;6(6):e70036. doi: 10.1002/bco2.70036 (PMC12116330; doi:10.1002/bco2.70036)
Supplement: Supplementary file 1 — Data S1. Implementation toolkit for the NICE Cambridge Prognostic groups and Predict Prostate [file BCO2-6-e70036-s001.pdf]

Informed decision making and evidence-based classification of men with newly diagnosed prostate cancer

Implementation toolkit for the NICE Cambridge Prognostic groups and Predict Prostate

Informed decision-making is critical in counselling men with newly diagnosed prostate cancer. While it is clear that men with poor prognosis locally advanced (CPG4-5) and metastatic disease need treatment, the question of the right option for those diagnosed with earlier-stage disease remains a dilemma.

Most patients still rely on their healthcare providers to guide them on treatment decisions, believing that professionals must know what the real risks and benefits are and use the most up-to-date information available. Hence when counselling men or giving information on options, a clinician's understanding of the individuals personal cancer and overall prognosis is imperative for discussing the value of treatment and then the outcomes of management.

This information must also be given without bias and with thorough knowledge and use of objective tools as recommended by national guidelines. The consequences of variations in clinician knowledge base and not using standardised tools is well documented in impact on treatment variations across the country, for example:

Thurtle *et al* 2019, https://www.ncbi.nlm.nih.gov/pmc/articles/PMC6889281/

Parry *et al* 2020, https://www.ncbi.nlm.nih.gov/pmc/articles/PMC7254634/

This tool kit provides a step by step guide on how to use the NICE recommended Cambridge Prognostic Groups and the NICE endorsed Predict Prostate tools for counselling and informed decision making for men with newly diagnosed non-metastatic prostate cancer. Also see **Figure 1** for a process pathway.

Further information and seminars on understanding prognosis and informed decision making can be found here : https://vimeo.com/showcase/cpc-cap-course

Guidance for practical implementation of nationally endorsed informed decision-making resources for prostate cancer

Standard of care

• **New cancer diagnoses and counselling must be done by a clinician with sufficient clinic time and interest to use the models and tools**. If this is not possible, the new diagnosis cases should be passed to a colleague who does have the time and interest. No patient should have a rushed clinic review when given a new diagnosis of prostate cancer.

• NICE guidance (NG131) lays out the principles for informed decision making : https://www.nice.org.uk/guidance/ng131

• The principles of shared decision making should be used in all circumstances https://www.nice.org.uk/about/what-we-do/our-programmes/nice-guidance/nice-guidelines/shared-decision-making

• The standard of care should be that the diagnosis of a new cancer is given by a senior clinician with CNS support (IOG guidelines on patient centred care, page 56 of - https://www.nice.org.uk/guidance/csg2/resources/improving-outcomes-in-urological-cancers-pdf- 773372413). Other alternative models of care should ensure that the health care professionals involved have a thorough knowledge of prognosis and disease natural history.

NICE Cambridge Prognostic Group (CPG) classification for non-metastatic prostate cancer

• All new diagnosis should be classified using the **NICE Cambridge Prognostic Groups (CPG)** in the MDT discussion and records, letters and all communications. The 25-year-old terminology of 'risk" (based on biochemical relapse after surgery or radiotherapy) is no longer relevant in the context of modern disease management. The NICE classification for prostate cancer was changed in 2021 and the rationale for this is outlined here :
https://www.nice.org.uk/guidance/ng131/evidence/evidence-reviews-for-risk-stratification-of-localised-prostate-cancer-pdf-10895771342

• **The CPG classification can be accessed at the NICE NG131 website**
https://www.nice.org.uk/guidance/ng131

or calculated at this website
https://cambridgeprognosticgroup.com

• CPG is now used by **all** prostate cancer charity literature and is also used by the NHS main information site https://www.nhs.uk/conditions/prostate-cancer/treatment/#:~:text=Prostate%20cancer%20can%20be%20categorised,group%20the%20prostate%20cancer%20is

• CPG has also been recognised in the **European Association of Urology 2024 guidelines** as being a superior prognostic stratification model compared to the old risk classification system

https://uroweb.org/guidelines/prostate-cancer/chapter/classification-and-staging-systems

- For men with likely <10-year Life expectancy, the ONS male life expectancy calculator is useful to contextualise any gain from treatment.

- Ideally **separate the diagnosis clinic from a treatment clinic** to avoid bias and influencing patient decision making.

- **Offer men access and a link to the "Know Your Options" webtool** developed by the NHS East of England Cancer Alliance. This is a link to NICE National Guidance on prostate cancer treatment options found at https://www.canceralliance.co.uk/prostate. This can be provided verbally, by text or in letters and/or by using the QR code below which can be pasted into letters (see exemplar letter 1 and 2 further below):

Prostate cancer: Knowing your options: A guide for patients to the National Institute of Health and Care Excellence (NICE)
recommended treatments for a new prostate cancer diagnosis - https://www.canceralliance.co.uk/prostate

Other resources available include this website and QR code that has a range of links to prostate cancer information webtools.

www.linktr.ee/prostate_cancer

This web-link tool can also be sent to patients mobile phones using the Accurx (www.accurx.com) NHS endorsed and secure platform

- **CPG1-3 DO NOT ASK FOR A TREATMENT DECISION** at the diagnosis visit. Encourage men to look at all information and consider for 48-72 hrs with family (or as much time as needed). Follow-up with CNS supported second discussion and final decision. NHS Targets should not rush a decision. There is no adverse prognostic effect by a 1-2-week delay in treatment.

- **CPG1-2 :** CPG1-2 disease is not counted in the Cancer Wait Time targets

- **Supplement the CPG information and national guidance with validated sources** e.g.
Cancer Research UK: https://www.cancerresearchuk.org/about-cancer/prostate-cancer
MacMillan : https://www.macmillan.org.uk/cancer-information-and-support
Prostate Cancer UK: https://prostatecanceruk.org/prostate-information-and-support

Men diagnosed with CPG1-3 and selected men with poorer prognosis disease but with significant co-morbidity or limited life expectancy.

In August 2019 NICE endorsed the use of the Predict Prostate prognostic tool as a decision aid to support its recommendations and quality standard for prostate cancer. NICE recommends its use as a tool for comparing the outcomes of conservative management and radical treatment, in men with non-metastatic prostate cancer:
(https://www.nice.org.uk/guidance/ng131/resources/endorsed-resource-predict-prostate-6898604077).

The European Association of Urology 2024 guidelines also now recommend using Predict Prostate in counselling and estimating treatment benefit versus active surveillance
https://uroweb.org/guidelines/prostate-cancer/chapter/classification-and-staging-systems

Predict Prostate (https://prostate.predict.cam) is an individualized multivariable clinical prognostic tool to contextualize a new prostate cancer diagnosis and the relative benefits of therapy against the metrics of both cancer-specific and overall survival. It considers both patient factors and clinicopathological features simultaneously when providing prognostic information at diagnosis. The underlying ethos is to provide unbiased information which is as bespoke as possible to give individual estimates of prognosis rather than categorized stratum.

To date Predict Prostate has been tested and validated in >350,000 men across 4 countries and in multiple ethnicities and age groups. The evidence based and published papers can be accessed from the webtool pages. Clinical use has been shown to reduce uncertainty on treatment choices amongst patients and variability in clinician recommendations. Its use has also been demonstrated to reduce over-treatment for early stage and favourable prognosis disease. The evidence for this can be read in the following papers :

Thurtle *et al* 2019, https://www.ncbi.nlm.nih.gov/pmc/articles/PMC6889281/

Thurtle *et al* 2021,
https://www.sciencedirect.com/science/article/pii/S0302283821019333?via%3Dihub

Pandiaraja *et al* 2024, https://bjui-journals.onlinelibrary.wiley.com/doi/10.1002/bco2.311

Implementing and using Predict Prostate in clinical counselling

• **Men diagnosed with CPG 1-3 disease** in which active surveillance or treatment is an option should have a Predict Prostate estimate calculated and documented in the MDT and subsequent diagnosis clinic communications.

• **Give access to the webtool to men and their families** with the information needed to populate it. The Predict Prostate tool is also incorporated in the https://www.canceralliance.co.uk/prostate website

- The text and link below can be included in letters so men can access Predict Prostate themselves. Use and interpretation should always be guided by a clinician

> **Predict Prostate** : Individualised prognostic model for men newly diagnosed with non-metastatic prostate cancer
> To estimate the relative treatment benefit for a new prostate cancer diagnosis: https://prostate.predict.cam

- The outputs from Predict Prostate can be printed out and/or relevant sections copied and pasted into the patient notes and letters as record of the discussion and information given. An example of how to use Predict Prostate outputs that in electronic notes & letters (see exemplar letter 1 and 2 further below):

(i) Prepare pre-set text in a template in letters/communications to patients & GPs e.g.

Estimated gain in survival from selecting immediate radical treatment compared to Initial management by monitoring and having treatment in future if needed

Based on Predict Prostate : https://prostate.predict.cam
At 10 years/ 15 years from diagnosis (*delete as needed*)

(ii) Cut and paste results from the Predict Prostate output (e.g. using the Text display)

91 out of 100 men are alive at 15 years with initial conservative treatment.
94 out of 100 men treated (an extra 3) are alive because of radical treatment.
Of the men who would not survive, 2 would die due to causes not related to prostate cancer.

Or using the icon display

This display shows the outcomes for 100 men. These results are based on the inputs and treatments you selected 10 15 years after diagnosis

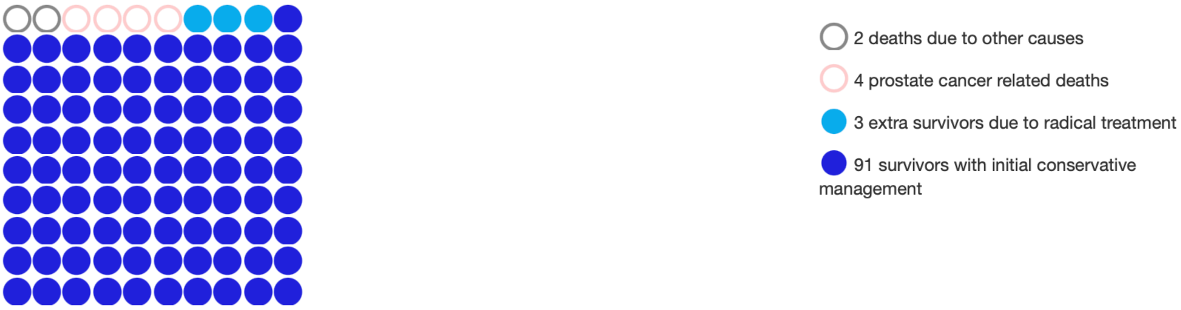

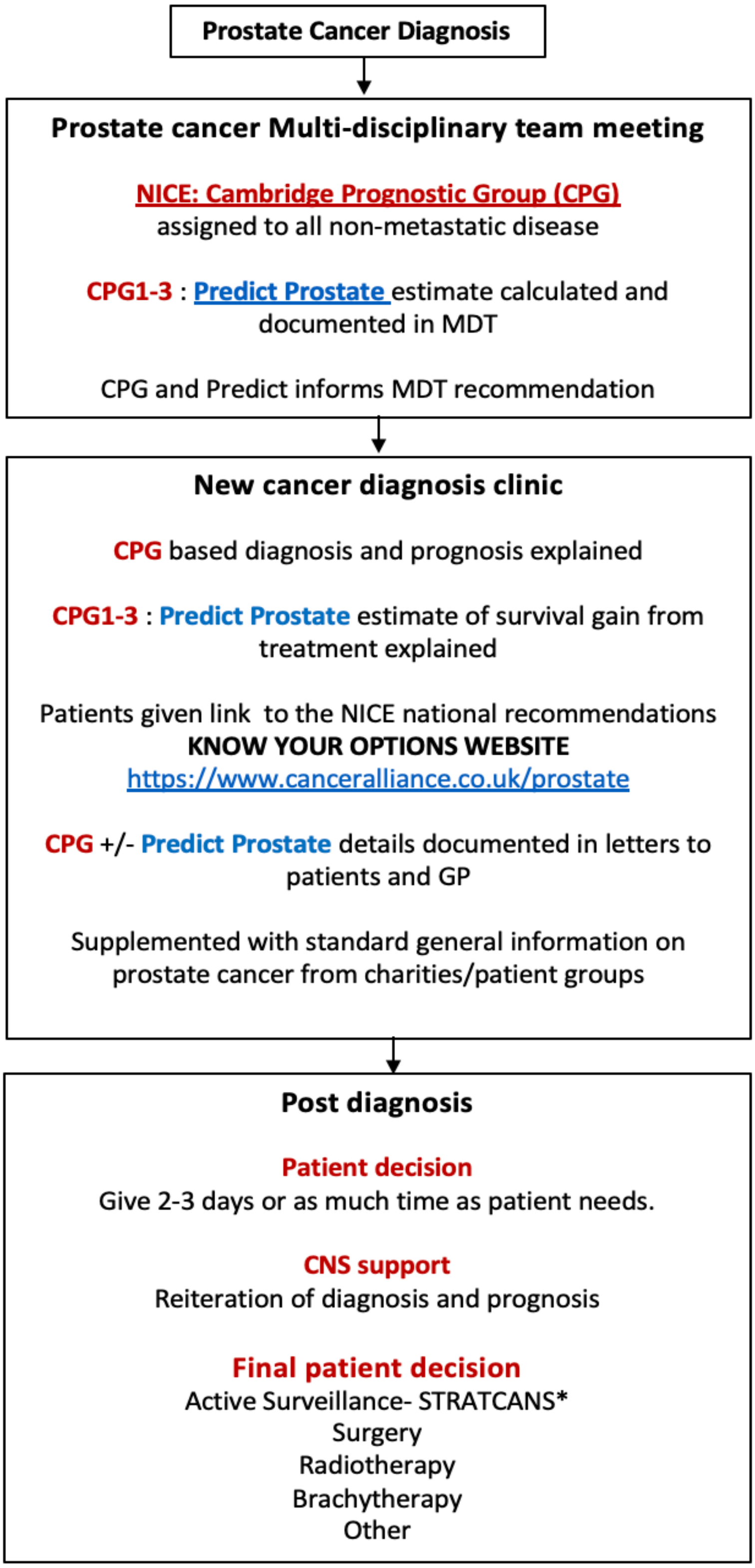

Figure 1 – Process pathway for a structured informed decision-making clinic for men with newly diagnosed prostate cancer. Also see implementation toolkit at https://stratcans.com/Implementation-Toolkit-NICE-CPG-Predict.pdf. *STRATCANS - Risk stratified active surveillance programme.

Exemplar clinic letter 1

Dear Doctor

DIAGNOSIS: **Prostate cancer diagnosis Dec 2023.**
PSA 5.4 MRI T2 PI-RADS 3, 52 cc
PSA density 0.10
Biopsies grade group 1 in 4/13 sites
Cambridge Prognostic Group 1

I reviewed this 71 yr old fit man in clinic and was pleased to hear he came through his biopsies well. These have come back showing evidence of prostate cancer with the above characteristics and I have today gone through the diagnosis and prognosis with him. In essence this would represent very favourable disease. In this case the primary recommendation is to consider active surveillance but he can also look at different treatment options. I have today briefly run through what these are and entail.

Estimated gain in survival from selecting immediate radical treatment compared to Initial management by monitoring and having treatment in future if needed

Based on Predict Prostate : https://prostate.predict.cam

At **15 years** from diagnosis

47 out of 100 men are alive at 15 years with initial conservative treatment.
51 out of 100 men treated (an extra 4) are alive because of radical treatment.
Of the men who would not survive, 46 would die due to causes not related to prostate cancer.

He has today received printed information and seen our nurse specialist and he can also access the national recommendations using the weblinks on this letter. We can see what his decision may be and take further management forward.

Links to national guidance on prostate cancer treatment options and Individualised decision aids

Prostate cancer: Knowing your options: A guide for patients to the National Institute of Health and Care Excellence (NICE)
recommended treatments for a new prostate cancer diagnosis - https://www.canceralliance.co.uk/prostate

Predict Prostate : Individualised prognostic model for men newly diagnosed with non-metastatic prostate cancer
To estimate the relative treatment benefit for a new prostate cancer diagnosis: https://prostate.predict.cam

Yours sincerely

Exemplar clinic letter 2

Dear Doctor

DIAGNOSIS: **Prostate cancer diagnosis Dec 2023.**
PSA 5.4 MRI T2 PI-RADS 3, 52 cc
PSA density 0.10
Biopsies grade group 1 in 4/13 sites
Cambridge Prognostic Group 1

I reviewed this 71 yr old fit man in clinic and was pleased to hear he came through his biopsies well. These have come back showing evidence of prostate cancer with the above characteristics and I have today gone through the diagnosis and prognosis with him. In essence this would represent very favourable disease. In this case the primary recommendation is to consider active surveillance but he can also look at different treatment options. I have today briefly run through what these are and entail.

Estimated gain in survival from selecting immediate radical treatment compared to Initial management by monitoring and having treatment in future if needed

Based on Predict Prostate : https://prostate.predict.cam
At **15 years** from diagnosis

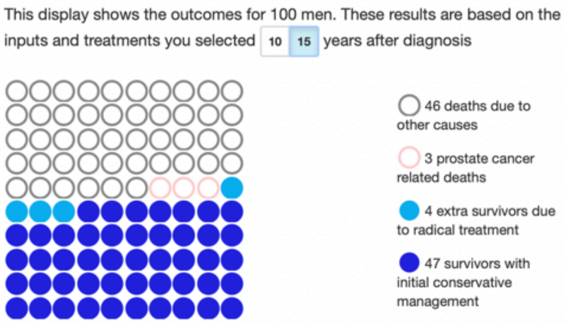

He has today received printed information and seen our nurse specialist and he can also access the national recommendations using the weblinks on this letter. We can see what his decision may be and take further management forward.

Links to national guidance on prostate cancer treatment options and Individualised decision aids

Prostate cancer: Knowing your options: A guide for patients to the National Institute of Health and Care Excellence (NICE)
recommended treatments for a new prostate cancer diagnosis - https://www.canceralliance.co.uk/prostate

Predict Prostate : Individualised prognostic model for men newly diagnosed with non-metastatic prostate cancer
To estimate the relative treatment benefit for a new prostate cancer diagnosis: https://prostate.predict.cam

Yours sincerely

References

EAU Prostate Cancer Guidelines (2024), https://uroweb.org/guidelines/prostate cancer/chapter/treatment

NICE Prostate Cancer Guidelines (NG131) https://www.nice.org.uk/guidance/ng131/resources/endorsed-resource-predict-prostate-6898604077).

NICE IOG guidelines on patient centred care, https://www.nice.org.uk/guidance/csg2/resources/improving-outcomes-in-urological-cancers-pdf- 773372413)

Parry, M G et al. "Risk stratification for prostate cancer management: value of the Cambridge Prognostic Group classification for assessing treatment allocation." BMC medicine vol. 18,1 114. 28 May. 2020, doi:10.1186/s12916-020-01588-9

Pandiaraja M, Pryle I, West L, Gardner L, Shallcross O, Tay J, Shah N, Gnanapragasam V, Lamb BW. Utilisation and impact of predict prostate on decision-making among clinicians and patients in a specialist tertiary referral centre: A retrospective cohort study. BJUI Compass. 2023 Nov 20;5(4):489-496. doi: 10.1002/bco2.311

Thurtle DR, Jenkins VL, Pharoah PD, Gnanapragasam VJ. Understanding of prognosis in non-metastatic prostate cancer: A randomised comparative study of clinician estimates measured against the PREDICT prostate prognostic model Br J Cancer (2019) IF 9.0 Oct;121(8):715-718.

Thurtle D, Jenkins V, Freeman A, Pearson M, Recchia G, Tamer P, Leonard K, Pharoah P, Aning J, Madaan S, Goh C, Hilman S, McCracken S, Ilie C, Lazarowicz H, Gnanapragasam VJ Clinical impact of the Predict Prostate risk communication tool in men newly diagnosed with non-metastatic prostate cancer: a multi-centre randomised controlled trial. Eur Urology. (2021) IF 24. Sep 4:S0302-2838(21)01933-3.
